# Environment and genotype predict the genomic nature of domestication of salmonids as revealed by gene expression

**DOI:** 10.1098/rspb.2022.2124

**Published:** 2022-12-07

**Authors:** James K. Bull, Brenna C. M. Stanford, Jessy K. Bokvist, Matthew P. Josephson, Sean M. Rogers

**Affiliations:** ^1^ Department of Biological Sciences, University of Calgary, Alberta, Canada T2N 1N4; ^2^ Fisheries and Oceans Canada, South Coast Area Office, Nanaimo, British Columbia, Canada V9T 1K3; ^3^ Bamfield Marine Sciences Centre, Bamfield, British Columbia, Canada V0R 1B0

**Keywords:** salmonidae, salmon, domestication, hatchery, gene expression, transcriptomics

## Abstract

Billions of salmonids are produced annually by artificial reproduction for harvest and conservation. Morphologically, behaviourally and physiologically these fish differ from wild-born fish, including in ways consistent with domestication. Unlike most studied domesticates, which diverged from wild ancestors millennia ago, salmonids offer a tractable model for early-stage domestication. Here, we review a fundamental mechanism for domestication-driven differences in early-stage domestication, differentially expressed genes (DEGs), in salmonids. We found 34 publications examining DEGs under domestication driven by environment and genotype, covering six species, over a range of life-history stages and tissues. Three trends emerged. First, domesticated genotypes have increased expression of growth hormone and related metabolic genes, with differences magnified under artificial environments with increased food. Regulatory consequences of these DEGs potentially drive overall DEG patterns. Second, immune genes are often DEGs under domestication and not simply owing to release from growth-immune trade-offs under increased food. Third, domesticated genotypes exhibit reduced gene expression plasticity, with plasticity further reduced in low-complexity environments typical of production systems. Recommendations for experimental design improvements, coupled with tissue-specific expression and emerging analytical approaches for DEGs present tractable avenues to understand the evolution of domestication in salmonids and other species.

## 1. Introduction

The evolution of animal domestication has had a pivotal role in the development of human societies. Yet, the genetic basis of domestication remains poorly understood. Conflicting theories of the genomics of domestication persist [1], focusing on either key traits (e.g. docility) and genetic variants underpinning them [1], or on changes in developmental pathways with diverse downstream consequences [2]. While convergent phenotypes, e.g. floppy ears in domesticated mammals [2], suggest convergent genomic evolution, this has been shown to occur only at higher genomic organizational levels. Few, if any, individual mutations are shared across domesticated species, while several genes and many gene networks have been identified to be involved in domestication across species [3]. Overall, it remains unclear if convergence occurs during domestication or owing to secondary selection after domestication, a distinction obscured by the time since domestication in most cases, e.g. 5000–8000 years for livestock [4].

**Figure 1.**
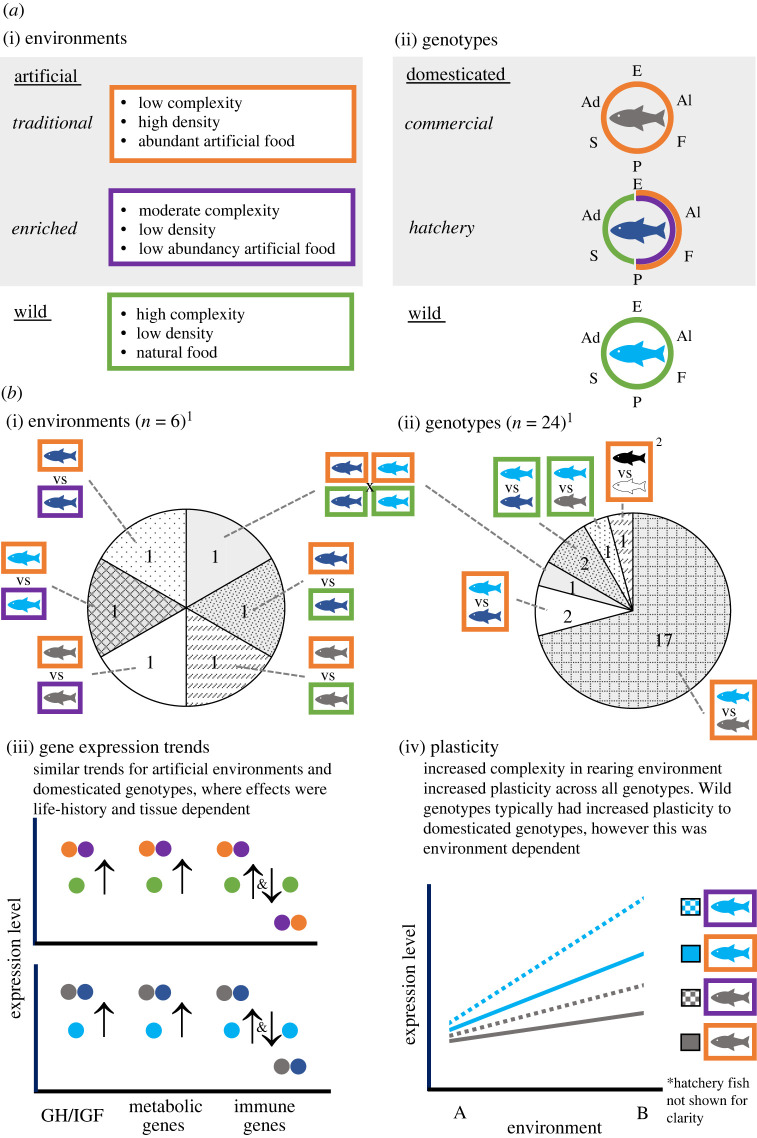
(*a*) Conceptual definitions of classes of (*a*(i)) environments and (*a*(ii)) genotypes as relevant to salmonids domestication. For genotypes, the portion of the life cycle exposed to different environments is shown (E, eggs; Al, alevin; F, fry; P, parr; S, smolt; Ad, adult). (*b*) Summary of studies included by environmental (*b*(i)) and genotypic (*b*(ii)) contrasts investigated. Our main findings with respect to (*b*(iii)) gene expression patterns and (*b*(iv)) plasticity. Colours of fish and boxes in (*b*) are as defined in (*a*). ^1^Sums to greater than the number of studies owing to one genotype-by-environment study included in both charts. ^2^Experimentally bred fish. Compared directionally bred (for growth) and randomly bred strains. GH, growth hormone; IGF, insulin-like growth factor. (Online version in colour.)

Contrastingly, the evolution of domestication in salmonids (family Salmonidae) is recent, on the scale of single to few-dozen generations [4,5], thereby offering a powerful opportunity to understand the early stages of underlying processes. While many aspects of the mammalian domestication syndrome appear different in fishes, parallels exist, such as greatly reduced antipredator responses, suggesting convergence in certain traits [6,7]. Although debate exists on requirements for fishes to be considered domesticated, ‘production’ salmonids, those produced by artificial fertilization and reared for at least part of their life in controlled environments, are among the candidates. Production salmonids may form populations with little geneflow shared with wild counterparts, may have life cycles that occur completely in artificial environments and have morphological, behavioural and physiological differences from wild fish [8] (figure 1). Data unambiguously indicate such differences have a heritable component [5,6,9,10]. For each of the approximately dozen commercially important salmonids, multiple domesticated strains exist, allowing the study of generalities and lineage-specific idiosyncrasies [11]. Although salmonids are not the numerically dominant domesticated group of fishes, they have been extensively studied owing to their socioeconomic and ecological importance.

### (a) Gene expression changes possibly underlie early domestication

Heritable phenotypes that diverge from wild phenotypes can appear rapidly under domestication in salmonids [10,12], including over a single generation [12]. The magnitude of such changes is often large. For example, weight at maturity, a common production trait, often increases by 10 to 20% per generation under directional selection [7]. This rapidity implies that *de novo* mutations that modify protein function are unlikely to underlie such effects [13]. Heritable differentially expressed genes (DEGs), which often segregate within populations [14], offer a potential mechanism [13]. DEGs are genes for which amounts of messenger RNA (mRNA) differ among individuals or in response to environmental variation, with mRNA amounts a proxy for quantity of the encoded proteins. Where DEGs are genes of known function, this can provide mechanistic insights [14], with changes in gene expression predicted to play crucial roles in rapid adaptive evolution and speciation [13,15]. Several analytical techniques exist for quantifying mRNA levels (table 1), with experiments typically examining DEGs between groups of interest or in relationship to variables (e.g. environmental) using standard ANOVA and regression analyses. Because proteins, in addition to their own functions, often interact with the function and regulation of other genes and proteins via interaction networks, DEGs in a small subset of genes can potentially have large phenotypic results [16]. For example, in mammals, differences in gene expression of a small number of genes in the brain have been linked to a range of key behavioural modifications in domestication [17]. Examining DEGs in salmonids allows investigation of whether DEGs seen in species at later stages of domestication [2] arise during early domestication. Oddly, directional selection appears to be unusually effective in salmonids [18], possibly owing to greater standing genetic variation because of recent domestication, or owing to extra evolvability resulting from ohnologues produced by the salmon-specific whole-genome duplication [19]. While salmonids differ from other domesticates in this respect, it is this fact that makes salmonids an ideal model for early stages of domestication where extensive standing genetic variation probably persists. Numerous studies have investigated gene expression differences between domesticated and wild salmonids, but only one has attempted to synthesize gene expression data, examining only a limited set of studies in Atlantic salmon (*Salmo salar*) [6]. Here, we review studies of gene expression differences under domestication across salmonids. Our goals are to advance the understanding of animal domestication by focused examination on a largely comparable set of studies and to make research recommendations.

**Table 1.** Basis, pros and cons of the three techniques ecological studies of gene expression have typically used to quantify mRNA levels. (All use complementary-DNA (cDNA) libraries prepared by reverse transcription of extracted RNA.)

technique	pros	cons
qPCR. Uses PCR and incorporates fluorescent probes or intercalating agents to quantify PCR product during amplification, with the number of cycles at which fluorescence exceeds background levels used as a measure of original mRNA amount. Stably expressed reference genes are used to standardize results	rapid	limited number of genes per study
	inexpensive	
	data analysis relatively simple	
microarray. Incorporates fluorescent dyes during reverse transcription and involve washing the cDNA mixture across slides dotted with 1000s of probes that selectively bind genes of interest. Fluorescence at these probes serves to quantify mRNA levels, typically in competition with a differently labelled reference pool for standardization	potentially quantifies genes genome wide	initial production of arrays requires existing genomic knowledge, limiting microarrays to commercially important species
RNAseq. Involves high-throughput sequencing of cDNA libraries, followed by bioinformatic processes to quantify the number of reads corresponding to each gene using internal standardization, e.g. number of reads for each gene per million reads	quantifies genes genome-wide	requires genomic and bioinformatic expertise to perform experiments and analyse data
	large dynamic range	
	does not require existing genomic resources	

## 2. Literature search

To locate relevant publications, all databases within the Web of Science were searched on 24 May 2022 using ‘AB = (Salmonidae OR Salmoninae OR Coregoninae OR Thymallinae OR Coregonus OR Prosopium OR Stenodus OR Thymallus OR Salmo OR Salvelinus OR Salvethymus OR Brachymystax OR Hucho OR Onycorhynchus OR Parahuco OR Salmon OR Trout OR Char OR Charr OR Steelhead OR Grayling OR Whitefish OR Lenok OR Taimen OR Nelma) AND TS = (captive* OR domestic* OR farm* OR hatcher*) AND TS = (microarray OR *expression OR RNA* OR *RNA OR transcript* OR qPCR OR exome)’, where AB requires the term to occur in the abstract and TS requires the term to occur in the title, abstract or keywords. The search was limited to English language material and returned 2405 results, of which 60 were discarded (four duplicates, 55 non-relevant books and book sections, and one patent). Abstracts for the remaining publications were read to determine if they met three criteria: (i) a member of Salmonidae was studied; (ii) there was a comparison between fish in traditional artificial- and wild- or enriched artificial environments (different environments; figure 1 for definitions), or between fish adapted to hatchery or farm environments and wild fish (different genotypes), or both; and (iii) gene expression was quantified; publications reporting semi-quantitative measurements (e.g. blotting techniques) were excluded. Comparisons involving transgenic fish were excluded, since gene expression is purposefully altered in these fish, although publications featuring transgenic fish were retained where comparisons among non-transgenic fish met the criteria above (e.g. [16]). Although environments were broadly classifiable, substantial differences exist within categories (e.g. different hatchery practices), which probably effect selection experienced by the respective strains.

Full texts of 109 retained publications were read to confirm all criteria were met, of which 33 did. As publications were read, citations were checked to locate additional relevant studies, but none were located. Finally, one publication was detected via a Web of Science alert, giving 34 publications in total.

### (a) Identified publications

The 34 publications report 29 unique studies and in five cases, different aspects (e.g. different life-history stages) of a study were reported in two separate publications (table 2). Across all studies, six species, all subfamily Salmoninae, were represented with Atlantic salmon accounting for half of all studies (table 2; key differences among species given in table 3). We classified domesticated strains into ‘commercial’, those subjected to directional selection for production traits and intended to differ from wild fish (e.g. farm fish) and ‘hatchery’, those only subjected to incidental selection and intended to be as similar to possible to wild fish, (e.g. hatchery fish produced using wild broodstock) (table 2). This distinction is further defined in figure 1. Throughout, we only make this distinction where relevant, otherwise using the general term ‘domestic’. For each study, we estimated number of generations the domesticated strain was under domestication and identified where stains were shared across studies from written descriptions (table 2; details given in the electronic supplementary material, table S1).

**Table 2.** Details of studies examining gene expression under domestication in salmonids. Studies including multiple tissues, life-history stages or analytic techniques are split over multiple lines. ‘?’ indicates value was not obtainable. Domestic strains were classified as commercial (directionally bred for production) or hatchery (not directionally bred for production). Details of estimation of number of generations, and identification of strains are given in the electronic supplementary material, table S1. Artificial test environments divided into (T) traditional and (E) enriched. Experiment type (type) indicates if domesticated and wild genotypes in a single environment (G) or a single genotype across environments (E) investigated. ‘MA’ indicates microarray in the methods column. no. tests refers to the number of microarray probes, genes or mRNAs tested. Species are Atlantic salmon (AS), brook charr (BC), brown trout (BT), chinook salmon (CH), coho salmon (CO), rainbow trout (RT) and steelhead trout (SH). Growth hormone/insulin-like growth factor (GH/IGF), immune genes (IMM) and plasticity (PLAST) indicate support for three broad patterns described in the main text. In each case, ↑ indicates an increase in the domesticated genotype relative to the wild genotype (or the artificial environment relative to the artificial enriched or wild environment), ↓ indicates the opposite, ↕ indicates mixed effects, ≈ indicates no effect and NA indicates no relevant data. Notes: 1: examined responses to crowding stress. 2: fish were a mix of wild and wild/domestic hybrids. Enhancement was the addition of gravel. 3: fish caught at sea as immature adults at three locations. Genotype composition of fish across locations differed. Examined DEGs in response to pollutants. 4: examined responses to several diets. 5: one domesticated strain, compared to two wild strains. 6: two domestic strains, each compared to its parental wild strain. 7: confirmation of microarray results. 8: examined response to aerobic training. 9: examined response to GH injection. 10: examined response to sea lice infection. 11: examined response to sediment stress. Genotype analysis includes hybrid fish. 12: one wild strain compared to two farmed strains. 13: compared directionally bred (for growth) and randomly bred strains. 14: wild-caught fish with different degrees of domestic heritage. Gene expression related to degree of domestic heritage. 15: expression measured at three time points. The latest is shown. 16: examined response to mock infection. 17: two hatchery stains compared to their wild originators. Only number of genes as DEFs in both comparisons are reported.

no.	species	domestic strain	strain	gens	test environment	LH stage	type	method	tissue	no. tests	no. DEGs	GH/IGF	IMM	PLAST	note and reference
1	AS	commercial	A	>10	artificial (T)	eggs	G	MA (44k)	whole body	31 491	165	NA	↓	NA	[20]
						fry	G	MA (44k)	whole body	30 164	2676	NA	↓	≈	1 [21]
2	AS	commercial	A	∼10	artificial (T)	alevin	G	MA (44k)	whole body	33 688	574	↕	↕	NA	[22]
						fry	G	MA (44k)	whole body	33 688	1038	↕	↓	NA	
3	AS	commercial	B	6	artificial (T) + artificial (E)	fry	E	MA (44k)	whole head	21 117	808	NA	NA	NA	2 [23]
4	AS	hatchery	?	?	wild	adult	G	RNAseq	liver	?	315 / 515 / 345	NA	NA	NA	3 [24]
5	AS	commercial	C	11	artificial (T)	fry	G	RNAseq	pyloric caeca	28 980	187	NA	NA	↑	4 [25]
									liver	24 119	379	NA	NA	↑	
6	AS	commercial	B	4	artificial (T)	fry	G	MA (16k)	liver	3715	32 / 39	NA	≈/≈	NA	5 [26]
7	AS	commercial	D / B?	7 / 5	artificial (T)	fry	G	MA (3.5k)	whole body	2552 / 3056	68 / 74	≈/≈	NA	NA	6 [27]
								qPCR	whole body	2	2	NA	NA	NA	7 [28]
8	AS	N/A	N/A	N/A	artificial (T) + artificial (E)	fry	E	qPCR	brain	1	1	NA	NA	↓	[29]
9	AS	commercial	A	∼10	artificial (T)	fry	G	qPCR	head kidney	7	7	↑	NA	≈	1 [30]
10	AS	commercial	E	∼10	artificial (T)	parr	G	RNAseq	heart ventricle	58 473	2515	NA	↓	↓	8 [31]
11	AS	N/A	N/A	N/A	artificial (T) + artificial (E)	parr	E	qPCR	brain	2	0	NA	NA	≈	[32]
12	AS	commercial	C	<11	artificial (T)	parr	G	qPCR	muscle	2	0 / 0	≈	NA	NA	9 [33]
									liver	2	0 / 0	≈	NA	NA	
									gill	2	0 / 0	≈	NA	NA	
13	AS	commercial	?	?	artificial (T)	smolt	G	qPCR	skin	20	3 / 4	NA	↓/↓	≈/≈	10 [34]
14	AS	commercial	B	4	artificial (T)	precocious males	G	MA (32k)	gill	4483	104	↓	↕	↓	11 [35]
15	AS	commercial	B / ?	∼7 / ?	artificial (T)	fry	G	MA (44k)+qPCR	whole body	12 828 + 8/5	46 + 3 / 200 + 3	NA	↕/↕	NA	12 [36]
16	BC	commercial	?	∼ 33	artificial (T)	alevin	G	MA (16k)	whole body	3263	276 / 265	NA	↕/↕	NA	5 [37]
17	BC	experimental	F	4	artificial (T)	alevin	G	MA (32k)	whole body	3481	0	NA	≈	NA	13 [38]
		(see note 13)				fry	G	MA (32k)	liver + pyloric caeca	2740	156	NA	≈	NA	
18	BC	commercial	N/A	N/A	wild	various	G	qPCR	liver	7	2	≈	NA	NA	14 [39]
									head kidney	2	1	NA	↓	NA	[40]
19	BT	hatchery	G	?	artificial (T)	parr	G	qPCR	spleen	8	3	NA	↕	NA	15 [41]
									head kidney	9	2	NA	↕	NA	
									liver	12	4	≈	↕	NA	
20	CH	commercial	?	?	artificial (T) + wild	fry	E	qPCR	liver	14	14	↓	↓	↓	1 [42]
21	CO	hatchery	H	∼8	artificial (T) + wild	smolt	GxE	MA (16k)+qPCR	?	? + 3	6 + 1	↓(E)/ ≈(G)	NA	NA [43]
22	CO	hatchery	H	∼10	artificial (T) + wild	fry	E	RNAseq	liver	?	5826	NA	↕	NA	[44]
23	CO	commercial	I	∼12	artificial (T)	parr	G	MA (16k)+qPCR	muscle	? + 11	78 + 7	↕	↑	NA	[16]
									liver	? + 11	274 + 3	↑	↑	NA	
								qPCR	muscle	21	15	↑	↓	NA	[45]
									liver	17	13	↑	NA	NA	
24	CO	commercial	?	?	artificial (T)	fry/parr (size match)	G	RNAseq	head kidney	?	6	NA	≈	↓	16 [46]
									liver	?	0	NA	≈	↓	
25	CO	hatchery	J / K	∼14	wild	adult	G	RNAseq	liver	25 246	3643	NA	↓	NA	17 [47]
26	SH	hatchery	L	1	artificial (T)	fry	G	RNAseq	whole body	?	723	↑	↑	NA	[5]
27	RT	commercial	M	>15	artificial (T)	parr	G	MA (16k)	liver	8919	277	NA	↑	NA	[48]
						fry/parr (size match)	G	MA (44k)	liver	9386	207	NA	↑	NA	[49]
						parr (age match)	G	MA (44k)	liver	9386	233	NA	↑	NA	
28	RT	commercial	M	>12	artificial (T)	parr	G	MA (16k)+qPCR	muscle	4565 + 4	398 + 4	NA	NA	NA	[50]
									liver	4886 + 4	269 + 4	↑	NA	NA	
									brain	7806 + 2	201 + 2	NA	NA	NA	
29	RT	commercial	M	>12	artificial (T)	parr	G	qPCR	pituitary	3	?	≈	NA	NA	[51]
									liver	3	?	↑	NA	NA	
									muscle	3	?	≈	NA	NA

**Table 3.** Life histories of species represented. Lifespan and age of sexual maturity (maturity) in years. Details from [52].

species			habitat	life history	lifespan	maturity
AS	Atlantic salmon	*Salmo salar*	anadromous	iteroparous	∼10	3–9
BC	brook charr	*Salvelinus fontinalis*	freshwater	iteroparous	20+	3+
BT	brown trout	*Salmo trutta*	typically freshwater	iteroparous	20+	1+
CH	chinook salmon	*Oncorhynchus tshawytscha*	anadromous	semelparous	2–9	2–9
CO	coho salmon	*Oncorhynchus kisutch*	anadromous	semelparous	2–5	2–5
RT	rainbow trout	*Oncorhynchus mykiss*	freshwater	iteroparous	∼11	2+
SH	steelhead	*Oncorhynchus mykiss*	anadromous	iteroparous	∼9	3+

Most studies (23) compared different genotypes in one environment, with fewer (six) comparing one genotype across different environments (e.g. artificial versus semi-wild), and one study comparing both in a combinatorial fashion (figure 1). Several studies investigated genotype-by-environment interactions with ‘environment’ varying over aspects other than rearing environment, e.g. in response to crowding [21]. Twelve tissue types were studied, with liver and homogenized whole body (for early life-history stages) well represented. All life-history stages were similarly represented, although only five studies included either smolt or adult stages. Fifteen publications used microarrays as their main investigatory technique, 12 used primarily quantitative polymerase chain reaction (qPCR) and seven used RNA sequencing (RNAseq), although some publications used multiple techniques as supplementary or confirmatory methods. Full breakdowns of studies by factors are given in the electronic supplementary material, table S2.

Generally, ‘wild’ fish were offspring of artificially spawned wild-caught parents, with eggs reared under artificial conditions, i.e. F_1_ domestic fish (but see [24,34]). This is a consequence of the need to standardize rearing environment to isolate effects owing to genetic differences (the focus of most studies). While this does not strictly control for parental effects, it helps remove one of the main mechanisms of maternal effects in salmonids, the ability of high-quality females to secure nests providing high-quality rearing environments [53]. More than half of publications were produced by two Canadian groups; R Devlin (nine publications) and L Bernatchez (nine publications), and strains focused on by these groups, e.g. St John River Atlantic salmon [27], represent in-depth examinations.

We describe and discuss the general trends observed. A formal meta-analysis is precluded by the nature of the DEG data. RNAseq approaches have dynamic ranges orders of magnitude greater than do microarray approaches [54], while qPCR focuses on genes selected based on *a priori* likely differences. Restricting analyses to microarray studies (the most common approach) leaves many individual levels of factors of interest (e.g. specific tissues) insufficiently replicated for analysis. Although many publications did not focus on the evolution of domestication, our review makes a strong case that the combined set of studies provides a meaningful insight into the consequences of captive salmon responding to artificial environments. While it is possible publication bias may have resulted in an inflated estimated of the extent of DEGs, we believe the diverse motivations of included publications means the overall patterns described are robust. Lists of DEGs and overrepresented gene ontology (GO) terms for each study are given in the electronic supplementary material, tables S3–S6. GO describes and classifies gene functions in standardized and hierarchical terms [55].

## 3. Broad patterns of gene expression differentiation under domestication in salmonids

Across studies, three broad patterns emerged (figure 1), the first two of which were previously noted for Atlantic salmon [6] and we confirm to be shared with represented salmonids.

### (a) Growth hormone and related metabolic genes are pervasively upregulated

Increases in growth hormone (GH) expression in domesticated fish was observed for Atlantic salmon [30], brook charr [37], coho salmon [16] and rainbow trout [51], with increases up to 400% [16]; similar patterns were observed for expression of the receptor (GHR). In vertebrates, growth rate is largely controlled by GH, which causes the liver to produce insulin-like growth factors (IGFs), and results in increased appetite, feed conversion efficiency, and stimulates muscle and skeletal growth [33,56]. Consistent with GH stimulating expression of IGFs [57], increases in expression of IGFs in the livers of domesticated fish were observed in coho salmon [45,51], Atlantic salmon [30] and steelhead [5]. The upregulation of GH-related genes was observed for both commercial (e.g. [16]) and hatchery (e.g. [5]) strains, indicating positive selection for growth in captivity even where it is not intentionally applied. This finding is consistent with observed selection on growth-related genes across domesticated species [58,59] and the existence of consistent phenotypic differences under domestication across fishes [60]. Across studies that quantified GH or IGF, five detected upregulation for domesticated genotypes relative to wild genotypes, one detected the opposite and four detected mixed or no effects (table 2), although interpretation of this last category is difficult as null results may be owing to limited statistical power and owing to interactions of GH expression with environment as discussed below.

In addition to genetic differences in GH production between domesticated and wild salmonids, GH expression levels are affected by environmental conditions [51,56]. For example, in chinook salmon, IGF-I and GHR were upregulated in a semi-wild environment compared to a hatchery environment, independent of domestication history [42]. Notably, both environments featured abundant food. Food availability is likely to be lower in true wild environments than in production environments [53], and IGF and GH levels have been linked to food intake [56]. Further, food availability is almost certainly less temporally and spatially variable in captive environments, meaning fish can maintain higher digestive capacity without incurring metabolic costs associated with larger organs when they are underused owing to temporary food scarcity [61]. Therefore, differences in food availability between artificial and wild environments need to be quantified to understand the implications for domestication potential. Typically, in food-limited environments, morphological differences between domesticated and wild salmonids diminish [44], and this has also been observed for DEGs [44]. Such environmental dependence of apparent degree of domestication has not been examined in other animals for DEGs, and hints investigations in feral mammals with extant ancestors may prove informative about domestication.

Increased GH levels probably modifies expression of other genes [33], as supported by comparisons of wild and transgenic coho salmon and rainbow trout that had the same genetic backgrounds other than GH expression being controlled by a strong promoter, both of which resulted in over 300 DEGs [16,48], similar to domesticated strains in the same systems. For GH-transgenic coho salmon, DEGs relative to a wild strain that were shared by a domesticated strain overwhelmingly (87%) occurred in the same direction [16], indicating DEGs caused by GH transgenesis and by domestication are largely concordant. Beyond GH and IGFs, many of the genes upregulated in both transgenic and domesticated fish are related to muscle development, metabolism and energy production. In addition, genes involved in muscle degradation can be strongly upregulated [45], indicating not only do domesticated fish develop muscle more rapidly but that muscle turnover is also higher [57]. In domesticated brook charr [37] and rainbow trout [49], upregulated genes were statistically over-represented for the molecular and biological process cell mitosis and cell/tissue structure using GO terms. Based on this, a large portion of DEGs between domesticated and wild salmon might be explained by increases in GH driven by strong selection for rapid development and large body size alone. Under such a model, DEGs observed are either second-order effects driven by higher GH levels and the effects this has on regulating other genes [62], or because they share regulatory elements with GH.

The potential key role of GH in overall DEG patterns is consistent with evidence that regulatory genes with above-average number of connections in gene networks are drivers of domestications in animals [3]. For example, in chickens DEGs between domesticated and wild strains interact with on average 15% more other genes than non-DEGs in known protein–protein interaction networks [3]. Knowledge of protein–protein interactions, not yet established for salmonids, will enable testing if similar phenomena occur in salmonids.

### (b) Immune functions are often differentially expressed genes under domestication

Domestication is expected to result in changes to immune systems of salmonids for reasons relating to, often intentional, selection for resistance to pathogens present in artificial environments and relaxed selection for resistance to certain pathogens owing to antibiotic use [53]. Such changes are further influenced by energy trade-offs and regulatory consequences under enhanced growth [62,63], with GH and IGF having stimulating effects on immune systems in fishes [62]. As these factors predict both enhanced and diminished immune function, and because historical pathogen exposures differ across populations [64], it is unsurprising that immune genes are often, but inconsistently, DEGs.

The term ‘immune response’ was one of the top 10 upregulated GO terms for certain domesticated strains of coho salmon [16] and rainbow trout [49], while genes involved in tissue repair were upregulated under domestication in a separate coho salmon strain [5]. However, the downregulation of immune genes was observed under domestication in a third coho salmon strain [45], as well as in Atlantic salmon [20,31], and brook charr [37], and was more common overall. Across all studies, three found upregulation of the immune system for domesticated genotypes relative to wild genotypes, six found the opposite and eight found mixed effects or no effects (table 2). Of these, only two studies found directional effects for both GH/IGF and for the immune system and in both cases were in concordance (both upregulated in [5], both downregulated in [42]). Downregulated genes under domestication included both genes involved in the innate immune system, as in the case of downregulation of major histocompatibility complex genes in domesticated brook charr [37], and genes involved in the adaptive immune system, as in the case of downregulation of immunoglobulin genes in domesticated Atlantic salmon [31].

One explanation for overall trends towards downregulation of immune functions, despite stimulating effects of GH, is energy-limited immune-growth trade-offs [63]. Although food intake of domesticated salmonids probably exceeds wild salmonids in their respective environments, domesticated fish display increased feed conversion ratio (weight gain per unit food ingested) indicating reallocation of energy does occur [45]. Curiously, positive effect of GH and IGFs on the immune system was largely determined for fish that were not selected for rapid growth [62], and evidence from GH-transgenic coho salmon indicates the relationship between GH and immune system may break down at elevated GH [65].

Immune stimulation studies offer further insight into the evolution of domestication [40,41]. For example, Kim *et al*. [46] exposed coho salmon to a viral mimic, a bacterial mimic and a control, and found more DEGs between the control and immune stimulation for wild fish (152) than for domesticated genotypes (18), with DEGs mostly upregulated under immune stimulation, indicating a reduced immune response in domesticated fish. This difference did, however, disappear when DEGs were restricted to known immune genes [46]. Similarly, Gallardi *et al*. [34] exposed Atlantic salmon to sea lice (*Lepeophtheirus salmonis*), and found gene expression patterns of commercial fish tended to group more tightly in multi-dimensional analyses than those of wild genomes, indicating less variability in immune response.

### (c) Gene expression plasticity is reduced in domesticated genotypes and artificial-rearing environments

Phenotypic plasticity occurs where a genotype produces different phenotypes under different environments, enabling population persistence under changing or unpredictable environments [66]. In principle, domestication may reduce plasticity as captive conditions are more predictable than the wild, favouring an optimum genotype with low plasticity to avoid incorrectly interpreting cueing stimuli [66]. For higher level traits (e.g. morphology), reduced plasticity is often observed in domesticated salmonids [6]. For gene expression, plasticity occurs for individual genes (expression levels of single genes) and for the whole transcriptome (number of DEGs). Previous research has suggested evolutionary adaptation to novel conditions, such as domestication, may both increase [66] and decrease [67] the extent of plasticity.

To date, only one study has performed a factorial genotype by environment design, i.e. domesticated and wild genotypes reared in both artificial and wild environments; however, they reported overall differences in gene expression between genotypes and not on the degree of plasticity [43]. Phenotypic plasticity has, however, been quantified across environmental variables other than rearing environment, e.g. in response to crowding [21], typically finding reduced gene expression plasticity for domesticated genotypes. Among such studies, one found increased gene expression plasticity for domesticated genotypes relative to wild genotypes, five found the opposite and four found mixed or no effects. Reduced plasticity was observed both at the individual gene level, such as observed smaller mean per-gene expression differences upon exposure to a sediment stress for domesticated Atlantic salmon genotypes than for wild genotypes [35], and whole transcriptome level, such as significantly fewer DEGs under aerobic exercise in a swim tunnel for domesticated Atlantic salmon genotypes than for wild genotypes [31]. Curiously, the three studies that examined crowding stresses [21,30,32] found inconsistent or no differences in DEG plasticity between fish of wild and domestic ancestry, and it is unclear if crowding stressors are different from other environmental stressors. In summary, these findings indicate reduced gene expression plasticity seen in other animal systems may be established early in the domestication [3], although additional confirmatory studies are required.

In addition to variation in the degree of plasticity between wild and domesticated genotypes, plasticity is rearing environment dependent, i.e. gene expression plasticity is itself plastic. For example, Wellband *et al*. [42] raised domesticated chinook salmon in traditional low-complexity artificial environments and in enriched high-complexity artificial environments, finding fish reared in the enriched environment displayed greater changes in gene expression under a confinement stress than fish raised under the traditional environment [42]. Similarly, wild Atlantic salmon raised under enriched artificial environments showed greater increases in the expression of *NeuroD1*, associated with neuroplasticity and learning in this species [29], than fish raised under traditional artificial environments, when repeatedly exposed to navigating a maze [29]. Given the apparent importance of plasticity in understanding DEGs under domestication in salmonids, we recommend authors carefully consider if experimental conditions match those under which they are trying to understand dynamics.

## 4. Differences between studies are partially explained by tissue, life-history stage and strain

Despite the general patterns discussed above, individual studies produced idiosyncratic results highlighting the importance of experimental design considerations including life-history stage and tissues choice.

### (a) Life-history stage

**Figure 2.**
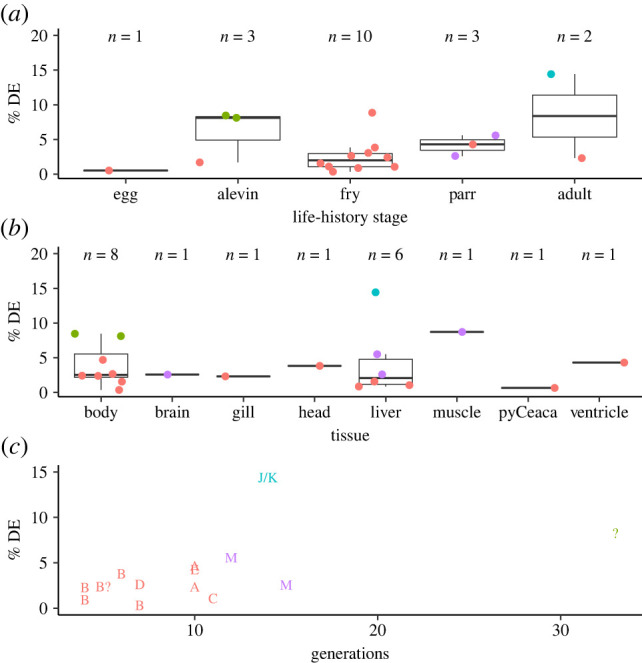
Per cent of genes/probes differentially expressed (DE) between domesticated and wild salmonid strains by (*a*) life-history stage, (*b*) tissue examined and (*c*) number of generations under domestication. Values averaged across tissues, life-history stages and/or strains as appropriate where multiple were examined in a study. Only studies using microarrays and RNAseq included, as qPCR—studies probably produce biased estimates owing to targeted selection of genes. For all plots, colours represent species, Atlantic salmon (red), coho salmon (blue), brook charr (green), rainbow trout (purple). For (*b*) ‘pyCeaca’ refers to pyloric caeca, ‘body’ homogenized whole body and ‘head’ to homogenized whole head. For (*c*) letters refer to strains as in table 2. (Online version in colour.)

Given the extended lifespan of most salmonids (table 3) and practical concerns with housing adults in experimental settings, the majority of studies examined early life-history stages. Whether these results inform domestication impacts at later life-history stages, e.g. interactions between wild and domesticated mature adults on breeding grounds [6] is unclear. Based on a review of six Atlantic salmon studies, Glover *et al*. [6] suggested that gene expression differences increase across the life cycle in salmonids, and this assertion is largely born-out, indicating estimates from early life-history stages probably underestimate differences in adult stages. Across studies, there is an upward trend in the median percentage of examined genes or probes differentially expressed between domesticated and wild genotypes across life-history stages (figure 2*a*). The exception, alevins, is drawn upwards by two estimates from brook charr, which has a much longer domestication history than other species represented (figure 2*c*). Additionally, two studies examined multiple life stages in a single experiment. These studies compared Atlantic salmon eggs and fry [20,21] and Atlantic salmon alevin and fry [22], reporting greater fractions of genes as DEGs in later life-history stages (0.5% versus 8.9%, and 1.7% versus 3.1% respectively). For [20,21], the possibility of more genes being expressed in later life-history stages driving this pattern can be discounted as only genes measurably expressed in both stages were included [20,21]. Enriched GO terms differed between life-history stages, indicating that different genes are DEGs at later life-history stages, not just a greater number [22]. Shifting terms reflected changes in developmental landmarks, from development-related pathways for eggs and alevin to digestion and metabolism as external feeding commenced for fry [22], highlighting the need for authors to select life-history stages relevant to the applications of their findings.

### (b) Tissue

Tissue-specific DEGs are common under domestication in salmonids. Only whole body and liver had multiple independent estimates of percentage of genes as DEGs and did not markedly differ (figure 2*b*). Several studies included multiple tissues, all comparing domesticated and wild genotypes in a single environment (table 2). Of these, Jin *et al*. [25] and Tymchuk *et al*. [50] are particularly informative as they survey genome-wide gene expression at a distinct life-history stage for multiple tissues.

Jin *et al*. [25] reared domesticated and wild Atlantic salmon on diets containing different lipid compositions and sampled pyloric caeca and liver for RNAseq. Across diets, they found more than twice as many DEGs in liver (379 of 24 119 genes) than in pyloric caeca (187 of 28 980 genes). Although they did not examine overlap at an individual gene level, only a single pathway from the Kyoto Encyclopedia of Genes and Genomes (KEGG [68]) was shared between the 11 and 17 pathways enriched in pyloric caeca and liver respectively, indicating strong tissue-specific effects. They found that these tissue-specific pathways largely reflected that organ's function, e.g. metabolic pathways in liver KEGG pathways. Tymchuk *et al*. [50] examined gene expression in brain, liver and muscle in domesticated and wild rainbow trout under artificial environments using a microarray and found that 3, 6 and 9% of probes were DEGs, respectively. Although several GO terms were shared by all (cytoplasm) or a pair of tissues (phosphate transport), the majority were tissue specific. One curious result of Tymchuk *et al*. [50] is that more genes were upregulated than downregulated under domestication in the brain (178 versus 23 DEGs), the opposite of the trend observed in most mammals and birds [3], possibly reflect the different history of salmonids and most domesticated animals. In other groups, docility is often assumed to be a first-evolved requirement for domestication, whereas in salmonids [1] initial selection may fall strongly on production traits with incidental selection for behaviours [8,9]. Currently, no further studies have been done that are able to confirm or refute this finding, and the brain deserves more attention, given its importance in understanding other systems [3].

### (c) Species and strain

Given the dominance of Atlantic salmon [6], we determined if Atlantic salmon differs systematically from other species. When compared to fish of the same life-history stage or tissue type, Atlantic salmon tended to have fewer DEGs (figure 2*a,b*), indicating that generalities derived from largely Atlantic salmon studies may underestimate the extent of DEGs for other species. However, as indicated by non-Atlantic salmon examples throughout, trends tend to be similar for other species. A potential explanation is that Atlantic salmon strains tend to have fewer generations under domestication that other species (figure 2*c*). Given that morphological differences accumulate with generations under selection [6,60], it is reasonable to hypothesize that DEGs may also do so. However, for both Atlantic salmon and for all species, this appears to only be true at the broadest scale (figure 2*c*).

It is possible trends are obscured by among-experiment variance. For example, both strains A and B have multiple estimates for given generations of domestication, and these estimates differ markedly (figure 2*c*). It is difficult to note the extent to which strains within species differ; however, we note no large differences among the five strains of Atlantic salmon for which percentage of genes as DEGs could be calculated, and extensive overlaps between DEGs under domestication is observed where multiple strains are included in a single study [27]. The large estimate of DEGs under domestication in hatchery Coho salmon [47] deserves comment. This was the only study to examine fully sexually mature individuals, and individuals that had completed the at-sea proportion of the anadromous life cycle. Given reproductive differences in the wild between domesticated and wild fish and associated evolutionary implications [10], confirmation of this finding is required.

## 5. Experimental considerations and future directions

### (a) Domesticated-wild strain matching

Salmonids exhibit extensive local adaptation [53], including in gene expression [27]. Where domesticated fish are compared to wild populations, some DEGs probably originate as differences between wild strains used and the wild ancestor of the domesticated strain [27]. This effect can be large, e.g. 6.8% of assayable genes occurred as DEGs between two strains of wild Atlantic salmon [27], while for each, less than 2% of assayable genes were DEGs when compared to their respective derived domesticated strain [27]. In the studies reviewed here, explicitly matching domesticated strains to their wild ancestors was rare, probably as many studies aimed to identify broadly how wild and domesticated fish differ to inform management (e.g. [37,51]), for which explicit matching may be unnecessary since domesticated strains are often farmed far from their origin [10]. Studies that aimed to look at domestication processes did, however, explicitly match domesticated strains to their wild ancestors (e.g. [5,32]). We are not suggesting that DEGs between wild and domesticated salmon in general result from this, but that caution should be taken when interpreting specific genes identified in experiments where domesticated fish are not the descendants of the wild strain used.

### (b) Multi-generational approaches

To date, no study has explicitly examined differences between domesticated and wild fish at more than one timepoint, i.e. at multiple generations of domestication, using the same sampling and analytical techniques for all time points. Such a study would probably be powerful for determining if domestication follows a specific order of event, e.g. do changes in gene expression in the brain leading to behavioural modifications [17] typically occur before metabolic differences? and for determining what genes are consistently differentially expressed in a system as opposed to stochastic generation-to-generation differences owing to genetic drift [11]. Such studies would be particularly informative for understanding differences seen between farm salmonids, where strains experience repeated selection over generations, and hatchery salmonids, where wild broodstock may be used every generation and thus selection does not ‘accumulate’ in the same way. Experimental and analytical frameworks for such an experiment have been demonstrated in model organisms [69] and the experiment of Sauvage *et al*. [38], where brook charr were directionally or randomly bred for four generations, indicates such an experiment is possible, at least in salmonids with short generation times.

### (c) Integrative approaches

In addition to certain knowledge gaps (brain tissue, late life-history stages, following certain strains over time), the field is sufficiently advanced to move beyond the generations of simple lists of DEGs towards more integrative studies. Notably, epigenetic mechanisms, which have strong theoretical bases for underlying gene expression differences [70], are now feasible to widely survey [44]. Several studies have begun to integrate gene expression, network analysis, epigenetic data and genome sequence data (e.g. [44]), increasing mechanistic understanding of domestication. While compiling studies, we were pleased to note open access to raw reads for all RNAseq studies. Given the likely future dominance of RNAseq, open access resources will form the basis of future formal meta-analyses. Although outcomes of RNAseq studies depend on experimental design and bioinformatic choices, there is value in providing certain intermediate files, particularly counts of reads per million bases per gene for all individuals in experiments, as recreating such files for meta-analysis will be computationally expensive. Providing such information was rare, and to our knowledge no current database is particularly well suited, although such files are typically small enough to be included as traditional supplemental material. Finally, several studies have demonstrated that investigation in the wild are possible by combining genetic estimates of the degree of introgression from domesticated strains and gene expression data [39,40]. In addition to their value as a model system, as human populations continue to grow, our reliance on domesticated salmonids as a food source will probably only increase [71], amplifying the need to understand domestication in these species.

## Data Availability

The data are provided in the electronic supplementary material [72].
